# Osteoporosis in the Elderly: A Cross-Sectional Study in Kazakhstan

**DOI:** 10.3390/ijerph22111694

**Published:** 2025-11-10

**Authors:** Aigul Abduldayeva, Gulnur Doszhanova, Saule Iskakova, Zhanar Bukeyeva, Saule Tarjibayeva, Yerkezhan Tolegenova, Ainagul Kazbekova, Olzhas Kozhamkulov, Aigerm Baimagambetova, Gulnaz Dosmyrzayeva

**Affiliations:** 1 Department of Research Institute of Preventive Medicine Named After Academician E. Dalenov, Astana Medical University, Astana 010000, Kazakhstan; abduldayeva.a@amu.kz (A.A.);; 2 Department of Internal Medicine No. 1, Astana Medical University, Astana 010000, Kazakhstan; 3 School of Public Health and Management, Astana Medical University, Astana 010000, Kazakhstan

**Keywords:** gerontological population, osteoporosis, osteopenia, actual nutrition, body mass index

## Abstract

The aim of this study was to assess bone health in individuals over 60 years of age in Kazakhstan, focusing on the relationship between osteoporosis, body mass index (BMI), body composition, and nutritional factors. This study included 1961 participants, consisting of 1620 women and 341 men, aged 60 to 89. Bone strength was assessed using quantitative ultrasound of the calcaneus, while fracture risk was assessed with the FRAX tool. Osteoporosis was detected in 20.2% of women and 15.2% of men, and osteopenia affected 59.8% of women and 58.4% of men. A total of 73.7% of the participants were overweight, 38.2% were pre-obese, and 35.5% were obese. The results of the study emphasise that, in addition to classic nutrients (calcium, vitamin D, protein), a number of trace elements and vitamins (selenium, iodine, zinc, vitamin B6, phytosterols) also play a significant, possibly indirect, role in bone metabolism. An inverse correlation was observed between BMI and osteoporosis prevalence; with a decrease in BMI, the incidence of osteoporosis increased (women: χ^2^ = 26.0, df = 2, *p* < 0.001; men: χ^2^ = 4.29, df = 2, *p* < 0.014; total sample: χ^2^ = 32.3, df = 2, *p* < 0.001), thus confirming that excess body fat exerts a protective effect on bone health. Significant risk factors for osteoporosis included age, height, and weight. A link was found between the age of first osteoporosis onset and BMI (from 65 to 72.14 years). This confirms the value of FRAX for accurately assessing fracture risk and developing personalised recommendations based on anthropometric and dietary characteristics. Future longitudinal research is warranted to validate these results and further elucidate the underlying mechanisms, including the predictive power of novel anthropometric parameters such as the Body Roundness Index and Body Shape Index.

## 1. Introduction

Osteoporosis (OP) is a major contributor to the high incidence of fractures and poses a major public health challenge in developing countries. Osteoporosis-related fractures result in disability, diminished quality of life, and premature mortality and impose a substantial economic burden. For example, in the United States, the annual cost of treating respondents with osteoporosis-related fractures is about USD 10 billion [1].

Osteoporosis affects 18.3% of the entire population. The interplay between serum 25-hydroxyvitamin D (25(OH)D) levels, dietary vitamin D intake, sun exposure, and bone mineral density (BMD) remains incompletely understood, despite the established influence of biochemical factors on fracture risk and the correlation between serum 25 (OH)D levels, sunlight exposure, and BMD [2].

In adults, vitamin D deficiency can negatively impact the development of osteopenia, osteoporosis, osteomalacia, and muscle weakness and increase the risk of fractures. Without a proper amount of vitamin D in the blood, only 10–15% of dietary calcium and approximately 60% of phosphorus are absorbed. A total of 33% of women aged 60 to 70 years and 66% of women aged 80 years and older are affected by osteoporosis [3,4]. It is estimated that 47% of women and 22% of men aged 50 and older will experience an osteoporotic fracture in their lifetime.

Osteoporosis has recently been classified as an age-related disease in people over 50 years of age. According to various researchers, its frequency ranges from 3 to 6% [5,6]. In 2014, 5.5 million men over the age of 50 were clinically diagnosed with OP in Europe [7]. In the future, it is expected that both the incidence of the disease and the number of fractures will increase. As of 2025, in Spain, 48% of all vertebral fractures are due to OP; 38% in France; 36% in Italy; 34% in Sweden; 33% in Germany; and 32% in Great Britain [8]. In men over 50 years of age, an epidemiological study conducted in Russia showed a high prevalence of fractures, as well as a further increase in the coming years [6].

According to the official request to the Ministry of Health of the Republic of Kazakhstan for 2023, the prevalence of osteoporosis among Kazakhstanis was 12.50^0^/_0000_, and the incidence was 4.23 new cases per 100,000 adults. Analysis of indicators of general and primary incidence of osteoporosis among the gerontological population group revealed a tendency of increase in the general incidence, from 26.69^0^/_0000_ in 2020 to 30.31^0^/_0000_ in 2023, while the primary incidence showed a downward trend from 10.70^0^/_0000_ in 2020 to 9.63^0^/_0000_ in 2023. The gerontological group in our study consisted of elderly and senile individuals, in accordance with the generally accepted organisation World Health Organization’s (WHO) and United Nations [9,10]. The incidence of osteoporosis naturally prevailed in women, with a value of 43.15^0^/_0000_ compared to men (10.57^0^/_0000_), and increased with age [9]. In Kazakhstan, scientific studies have been conducted to study the prevalence of osteoporosis and risk factors in specific geographical regions, such as the Abay district and the city of Taldykorgan [11,12]. The primary strength and distinctive characteristic of our study is the evaluation of the overall prevalence of osteoporosis-related conditions among the population aged over 60 years residing in a single geographical region of Kazakhstan—the Northern Region. This region (comprising the Akmola, Pavlodar regions, and the city of Astana) is distinguished by analogous climatic and demographic characteristics, with the collective population accounting for approximately 20% of the country’s total gerontological population [13].

The scale and representativeness of the data obtained allow for the reliable estimation of the prevalence of osteoporosis and its associated alimentary risk factors in the Northern Region. This is of fundamental importance for the development of targeted regional prevention programmes.

## 2. Materials and Methods

### Study Design, Sample, Population

This study employed a cross-sectional design that surveyed people aged 60–89 years, with an average age of 68.4 ± 5.1 years.

The study was conducted from 8 January 2025 to 3 August 2025, drawing upon data from several primary healthcare organisations: City Polyclinics No. 5 and No. 7, and the Republican Center for Primary Health Care (Astana), Polyclinic No. 3 (Pavlodar), and City Polyclinic (Kokshetau). The calculation of the required sample size to ensure the study’s power was performed using the online calculator sample-size.net. According to data from the study by Madiyeva, M. et al. [11], the expected prevalence of conditions associated with reduced bone mineral density is 48.3%. In order to assess the prevalence of osteoporosis with an accuracy of 5% (with a confidence interval length of 10%), it was sufficient to enrol 384 participants. A total of 1961 respondents were involved in the study, which significantly exceeds the calculated minimum and were selected from three regions (Akmola, Pavlodar regions, and the city of Astana). The distribution of survey respondents across each region was uniform, thereby ensuring the statistical robustness of the data.

The sample distribution by gender was 82.6% female and 17.4% male. The observed predominance of women, although statistically significant, correlates with the higher proportion of women in the general population of the gerontological cohort, reflecting the objective demographic situation.

The sample included 89.1% elderly people (60–74 years) and 10.9% senile people (75–89 years). The sample was selected using a simple probabilistic sampling method, with participants chosen randomly from a polyclinic registry using a random number generator. With over 1900 responses, a representative sample was guaranteed, as this would be sufficient to assess the prevalence of osteoporosis among the elderly population of the Northern Region of Kazakhstan, with a 95% CI and error ±5% [14].

Inclusion criteria were as follows: persons aged 60–89 years, absence of spinal surgery performed within the last six months; absence of inflammatory spinal diseases diagnosed or currently in the acute exacerbation phase within the last six months; and provision of informed consent to participate.

Exclusion criteria of this study were as follows: less than 60 years of age, older than 89 years, category I–III disabled groups, severe concomitant diseases, cancer and infectious diseases, congenital musculoskeletal disorders.

This study is the initial stage of a grant project scheduled for implementation in 2026–2027.

## 3. Research Variables

### 3.1. Quantitative Ultrasound Examination of the Calcaneus

Quantitative ultrasound of the calcaneus (densitometry) was conducted to assess bone health.

This method was chosen due to its accessibility and safety for large-scale screening. The strength characteristics of bone tissue in the respondents were evaluated by ultrasound calcaneal densitometry (SONOST 3000 densitometer, OsteoSys Co., Ltd., Seoul, Republic of Korea) using the *T*-test. According to this method, the normal values of T-criteria range from −1 to 2.25 standard deviations (SD), from the mean bone strength of adults of the same sex aged 50, when peak bone mass is typically achieved. Values from −1 SD to −2.5 SD refer to osteopenia, and values below −2.5 SD are classified as osteoporosis (OP). In the reference curves, considering modern densitometers, the deviation of −2 SD (*T*-test) corresponds to approximately 80% of the peak bone mass [15].

In Kazakhstan, calcaneal densitometry is used as an effective, non-ionizing radiation and user-friendly screening method to assess the condition of the skeleton and consequently diagnose the risk of osteoporotic fractures. The portability of the ultrasound device means that it can be used for household surveys, covering both urban and rural populations. In addition, considering the presence of metal implants in many elderly people, ultrasound was the optimal method for this group of respondents. Follow-up studies are expected to expand the scope of this study to include dual-energy x-ray absorptiometry as a more precise indicator of bone mineral density in at-risk participants.

The leading risk factors for OP development were measured by the one-minute osteoporosis risk test, a specialized questionnaire that evaluates general morbidity, as well as a 24 h nutrition replay test. Participants’ nutrition was reported by a weight questionnaire, which is advised by the WHO epidemiological studies [16,17]. FRAX was measured using an accessible web-based fracture risk assessment tool available on the website of the University of Sheffield, which included three risk categories of low risk, (<10%) moderate risk (10–20%), and high risk (>20%).

### 3.2. Analysis of Actual Nutrition

Data collection procedures emphasized detailed assessments of actual nutrition. Participants completed the 24 h dietary recall method recommended by the WHO on typical weekdays during the spring, summer, and autumn periods [18]. This approach enabled the researchers to document specific food items, portion sizes, meal frequencies, and cooking methods. Additionally, a frequency analysis of overall dietary patterns was carried out to identify common consumption habits for specific food groups. The information gathered through both techniques was then compared with recommended dietary intake norms for the population of the Republic of Kazakhstan.

To calculate the chemical composition of the diet and the energy of the consumed foods, a database with the chemical composition and nutritional value of products was used [19]. National recommendations, which were based on standards set by the WHO, were used as reference values for the assessment of energy and essential nutrient requirements. The Food and Agriculture Organization of the United Nations (FAO) scale was utilised for the assessment of micronutrients [20].

The nutritional status of the respondents, as an indicator of the impact of diet, was assessed using anthropometric measurements and the calculation of the Body Mass Index (BMI) for both sexes according to the following formula: *BMI = m/h^2^*, where *m* is body weight in kilograms, *h* is height in meters [14].

## 4. Data Analysis

IBM SPSS Statistics version 29.0 for Windows was used to perform the analysis. Descriptive statistics, ANOVA, and Pearson correlation tests were applied. The independent sample t-test was also used, and a *p*-value of less than 0.05 was considered statistically significant. The correlation was considered “weak” at coefficients less than 0.39, “moderate” at values from 0.40 to 0.59, and “strong” at coefficients greater than 0.6. Continuous variables are expressed as the mean standard deviation, and qualitative variables are expressed as *n* (%). Differences between the mean values of variables for each gender were calculated using an independent Student *T*-test. The Kaplan–Meyer test was used to calculate the early survival time.

## 5. Ethical Considerations

This research was conducted in accordance with the Kazakh legislation and the principles of the Helsinki Declaration [21]. The decision to participate in this study was made by each respondent independently and voluntarily. Respondents could also consult with another doctor about this study prior to participation. The respondent would be able stop participating in this study with no further questioning, and this study did not affect their further treatment. Participation in the research work was confidential. All participants gave their voluntary written informed consent to participate in this study.

## 6. Results

**Figure 1:**
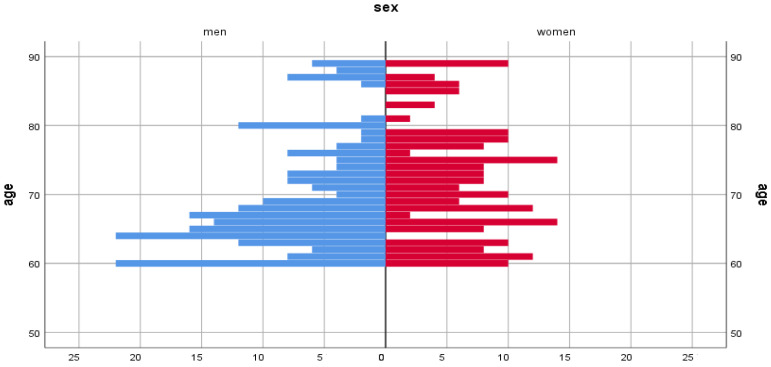
Gender and age composition chart.

To achieve a high-quality result, all respondents were distributed by gender and age (Figure 1).

The health status of elderly people was studied based on the assessment of bone strength characteristics, nutrition and body mass index, followed by the development of the FRAX model.

The survey results indicated inadequate consumption of foods rich in calcium and vitamin D, such as fish, fermented dairy products, cottage cheese, and cheese. Cottage cheese was said to have been consumed often by 28.9% of participants, rarely by 59.3%, and not at all by 11.8%. Cheese was consumed often by 29.4%, rarely by 60.7%, and not at all by 9.9%. Fermented dairy products were consumed often by 39.4%, rarely by 52.4%, and not at all by 8.2%. Among fermented dairy products, 18.0% of respondents consumed fermented baked milk, 14.0% consumed yogurt, 17.7% consumed koumiss, 11.1% consumed porridge, and 10.1% consumed kefir.

In terms of carbohydrates, respondents consumed more white bread and flour products than cereals, vegetables, fruits, berries, and nuts. Rye bread was practically absent from the diet. The incidence of sweets and caffeinated beverages was high. Black tea was consumed 3 to 4 times a day by 71.7% of respondents, 1–2 times a day by 17.8%, once a day by 6.9%, and no more than 2–3 times a week by 3.6% of respondents. A total of 71.3% of respondents often drank tea with milk, 17.4% rarely added milk, and 11.2% of respondents did not add milk at all.

Table 1 depicts how the chemical composition of foods affects bone metabolism and the consequent development of OP.

The caloric content of food was 1800.2 ± 242.3 kcal for women and 1820 ± 322.2 kcal for men. However, the current standard of living and amounts of physical activity caused a decrease in the total amount of caloric content. Additionally, age plays a role in decreased basal metabolism and physical activity, further influencing caloric intake.

The results of the correlation analysis revealed a statistically significant relationship between changes in the structure of bone tissue and the consumption of several foods. A moderate positive correlation was found between changes in bone structure and the following nutrients: total protein (r = 0.538, *p* < 0.01), phytosterols (r = 0.576, *p* < 0.01), and zinc (r = 0.575, *p* < 0.01). A strong positive correlation was found in selenium (r = 0.630, *p* < 0.01), iodine (r = 0.609, *p* < 0.01), vitamin B6, (r = 0.630, *p* < 0.01) and calcium (r = 0.713, *p* < 0.01). This indicates the importances of the aforementioned nutrients in maintaining bone structure and preventing osteoporosis among the elderly.

The analysis of variance revealed significant differences by sex in the consumption of animal proteins (F = 8.055, *p* = 0.005), nitrogen (F = 7.254, *p* = 0.008), vitamin C (F = 6.904, *p* = 0.010) and fibre (F = 6.358, *p* = 0.013), which emphasizes the need to account for gender differences in assessing the effect of these substances on bone health, especially among the elderly.

**Figure 2:**
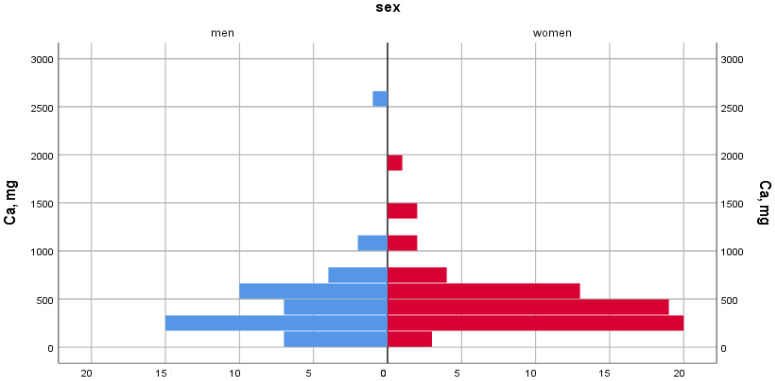
Proportion of people with calcium deficiency.

**Figure 3:**
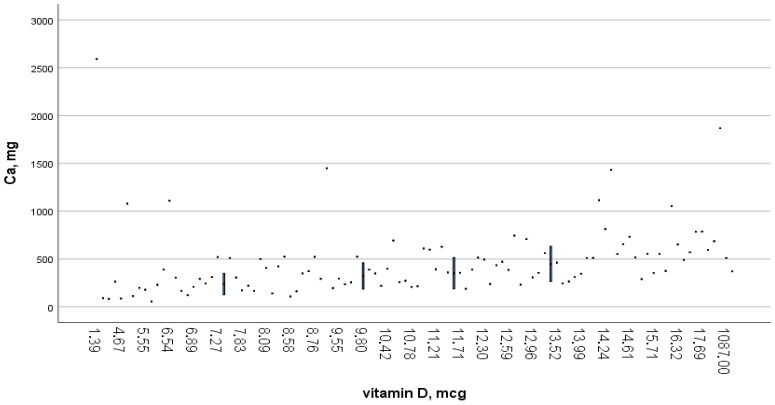
Calcium content depends on vitamin D content.

A total of 90% of women and men experience Ca deficiency due to a lack of vitamin D in foods (Figure 2 and Figure 3).

Dietary calcium intake was 528.9 ± 308.2 mg/day in women and 549.6 ± 353.5 mg/day in men, when the recommended norm is 1200 mg/day. In terms of dietary density, calcium intake was 329.9 mg/1000 kcal for women and 298.9 mg/1000 kcal for men. The proportion of people with nutritional calcium deficiency was 80.6% among women and 80.1% among men.

Figure 3 shows a scatter plot illustrating the relationship between vitamin D and calcium intake. As can be seen from the graph, the individual observations (data points) show a wide range of calcium consumption (mg) at different levels of vitamin D (mcg). To facilitate statistical summarisation, vertical blue lines have been included to denote the median and interquartile range of calcium intake within the specified vitamin D intervals.

At the recommended food density of 2.5–5.0 mg per 1000 kcal, vitamin D intake was 0.92 mg for women and 1.15 mg for men. The proportion of people with dietary vitamin D deficiency was 98.4% among women and 96.5% among men (Table 2).

Calcium deficiency disrupts the acid–base balance, leading to potassium deficiency, which amounted to 2681.1 ± 1252 mg per day in women and 2835.8 ± 1319.1 mg per day in men, which are lower than the recommended FAO value (3500 mg/day).

The identified nutritional imbalances contribute to metabolic disorders, including obesity, atherosclerosis, diabetes mellitus, hypertension, gastrointestinal disorders, kidney diseases, cancer, osteoporosis, and other pathological conditions.

**Figure 4:**
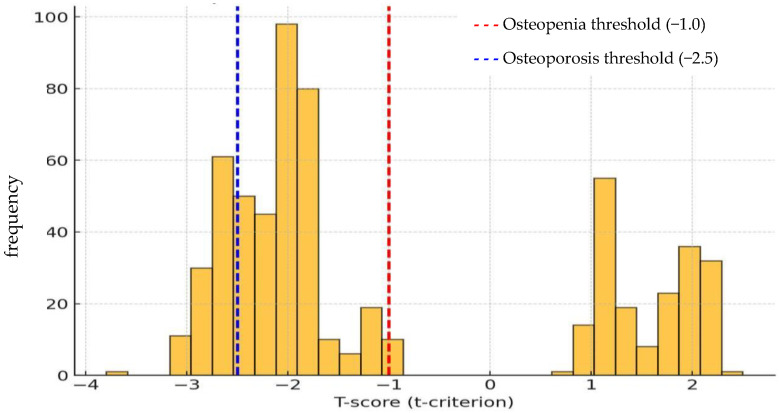
Distribution of T-score among study participants.

When assessing the strength characteristics of bone tissue in respondents, the Speed of Sound value was set to 1401 ± 15 m/s and Broadband Ultrasound Attenuation to 63 ± 25 dB/MHz. A decrease in the *T*-test was observed in 73.6% of men and 80.0% of women, which confirms the high prevalence of a decrease in bone mineral density in this age group (Figure 4).

Osteoporosis was diagnosed in 19.4% of individuals, and osteopenia in 59.5%. A total of 20.2% of women were diagnosed with OP, and 59.8% with osteopenia. Among men, osteoporosis was detected in 15.2% of cases and osteopenia in 58.4%. An analysis of bone strength characteristics by age revealed the following trend: in the 60–69 age group, 37.1% of individuals met the criteria for osteoporosis, while 48.7% showed signs of osteopenia. In the age group of 70–79 years, 50.8% of cases corresponded to OP and 38.6% to osteopenia. Among 80–89-year-olds, 80.8% were diagnosed with osteoporosis and 19.2% with osteopenia. The data obtained indicate a pronounced age dependence of the prevalence of osteoporosis and osteopenia (*p* < 0.001). With increasing age, there is an increase in the proportion of people with osteoporosis and a decrease in the proportion of people with osteopenia.

**Figure 5:**
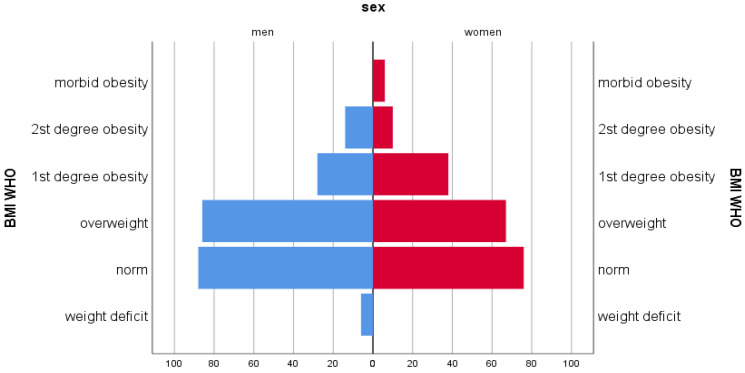
BMI distribution by gender.

Among the 1961 examined individuals, 696 (35.5%) were diagnosed with varying degrees of obesity. In terms of gender, 37.8% of women and 24.7% of men were found to be obese (Figure 5).

Of the participants, 73.7% were overweight, including 38.2% in the pre-obese stage, 22.8% with obesity, and 9.1% with II-degree and 3.6% with III-degree obesity.

Upon normal sonographic parameters, the average BMI reached 29.91 ± 0.29 kg/m^2^, osteopenia was 28.57 ± 0.16 kg/m^2^, and OP was 27.76 ± 0.29 kg/m^2^. In participants’ BMI distribution based on sonographic indicators for normal bone strength, OP, osteopenia, and gender–age classifications, an increase in BMI was statistically associated with a significant decrease in the detection of bone strength impairments. This trend was also observed across different gender and age groups (Table 3).

The data in Table 4 show a tendency for the average BMI to decrease as the condition of bone tissue worsens, which may indicate a link between body weight and bone health. A high coefficient of determination (R2 = 0.9997) indicates that the model almost perfectly explains the variability of data, emphasizing the significance of this relationship.

In all groups, women’s BMI was higher than men’s BMI, but in both groups, there was a tendency of lower BMI in respondents with OP (Table 5).

According to the data obtained, the detection rate of OP sharply decreased with an increase in body mass index (Table 6). With a decrease in body mass index, the frequency of OP detection increased (women—χ^2^ = 26.0, df = 2, *p* < 0.001; men—χ^2^ = 4.29 df = 2, *p* < 0.014; full group—χ^2^ = 32.3, df = 2, *p* < 0.001).

Regarding the prevalence of overweight and obesity, the data are almost comparable with the general group of respondents; i.e., the presence of OP does not mean the absence of excess fat mass, just as the presence of obesity does not exclude the presence of OP. The only proven fact is a greater tendency of damage to the strength properties of bone tissue at low body weight (χ^2^ = 14.9, *p* < 0.05).

**Figure 6:**
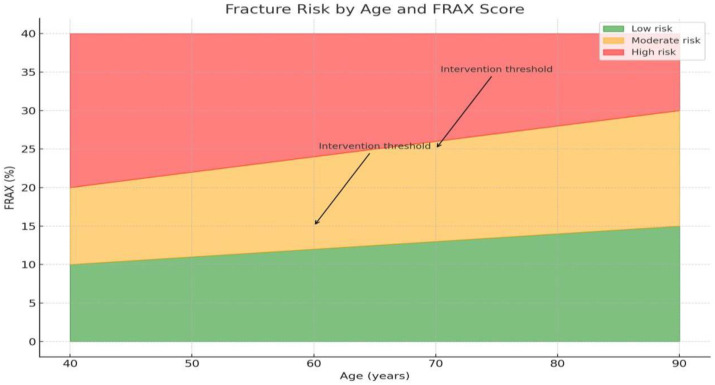
FRAX by age in Kazakhstan (arrows indicate threshold levels).

For a more accurate assessment of the individual risk of osteoporosis-related fractures, we developed the FRAX model (Figure 6). The use of this tool allowed us to establish that, despite considering anthropometric parameters and dietary characteristics, the leading risk factor for fractures is age.

**Figure 7:**
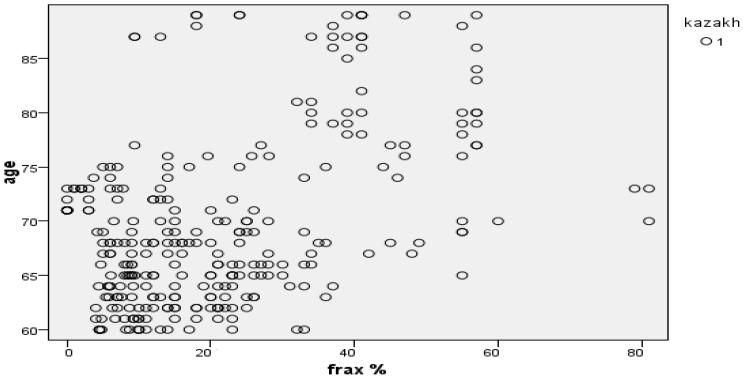
Fracture risk density in Kazakhs.

Figure 6 shows the distribution of respondents with osteoporosis by 10-year risk of major osteoporotic fractures and hip fractures calculated using the FRAX tool. According to data obtained in Kazakhstan, the risk of fractures among the population is 23.9 ± 0.65, which indicates a high risk of osteoporotic fractures in this population. Increased FRAX values were recorded in all senile individuals examined, which once again underlines the significance of the age factor in increasing the risk of osteoporotic fractures (r = 730.673). Figure 7 shows a high distribution density of fracture risk in Kazakhs aged 60–89 years.

In a survival analysis, it was found that the time of onset of osteoporosis varies depending on BMI. Respondents with low weight and morbid obesity develop osteoporosis at an average age of 65 years; in people with normal weight, this value is 69.47 years, with 71.15 years for overweight and 72.14 years for obesity. Thus, the use of FRAX allows us to more accurately stratify the risk of fractures and develop personalized recommendations for the prevention and treatment of osteoporosis, taking into account both anthropometric and dietary characteristics of respondents.

## 7. Discussion

This study is dedicated to the prenosological diagnosis of the health status of the elderly and senile population of Kazakhstan, based on the investigation of bone system condition and fracture risk, anthropometric data (including BMI), and actual nutrition.

The significance of this study lies in its identification of the relationship between anthropometric variables and key nutritional indicators that contribute to osteoporosis in Kazakhstan’s elderly population, with the goal of preventing fractures and highlighting the most significant indicators affecting these processes. The central question is to what extent the projected increase in life expectancy will be accompanied by improvements in quality of life, living conditions, and the duration of a healthy working life, ultimately contributing to ‘successful aging’ among Kazakhstanis [22,23]. According to the data obtained, poly-micronutrient deficiency was observed. There was a high intake of simple carbohydrates found in refined foods, such as sugar, flaked cereals and fine flour, while there was a low intake of dietary fibre obtained from fruits, vegetables, root vegetables, berries and other horticultural products. Such dietary features can negatively affect the balance of nutrients needed to maintain and increase bone density [24].

The results obtained from the correlation and dispersion analyses convincingly demonstrate a statistically significant relationship between the consumption of specific nutrients and changes in bone structure. The discovered association is consistent with the fundamental role of nutrition in maintaining skeletal health. Protein plays a fundamental role in the synthesis of the bone’s collagen matrix. Systematic reviews and meta-analyses confirm that adequate protein intake (especially in the elderly) is associated with improved BMD and a reduced risk of fractures [25,26,27]. The presence of significant differences in the consumption of animal proteins and, consequently, nitrogen (which is a marker of overall protein intake) reflects global dietary trends. Protein metabolism and requirements may differ between men and women, particularly following menopause. The work by M. Isanejad et al. showed that the protective effect of high protein intake on BMD or fracture risk may be more pronounced, or at least mechanistically distinct, in older men and women [28]. Women may be more susceptible to bone mass loss under conditions of low protein intake due to underlying hormonal changes. Our results demonstrate a moderate positive correlation with zinc (r = 0.575, *p* < 0.01) and a strong positive correlation with selenium (r = 0.630, *p* < 0.01) and iodine (r = 0.609, *p* < 0.01). Literature data, including analyses published in recent years, confirm that low zinc levels are associated with lower BMD, and its adequate consumption can stimulate osteoblastogenesis while inhibiting osteoclastogenesis [29,30]. Selenium and iodine are traditionally linked to antioxidant defence and thyroid function, respectively. However, as our results indicate, these trace elements exhibit a strong correlation with bone structure. Recent studies increasingly suggest that thyroid dysfunction, which is regulated by iodine, exerts a direct impact on bone metabolism [31,32]. Furthermore, the antioxidant role of selenium is crucial for reducing oxidative stress, a key factor contributing to osteoporosis and the deterioration of bone microarchitecture [33,34]. Thus, the strong correlation of these elements may be due to their indirect regulatory effect on bone remodelling. Vitamin B6 serves as a cofactor in homocysteine metabolism. Elevated homocysteine levels are known as an independent risk factor for osteoporosis and fractures, as they negatively affect the bone’s collagen matrix [35,36]. Our strong positive correlation may reflect the protective role of B6 in lowering homocysteine levels, thereby indirectly improving bone structure. Studies by D. Kim et al. indicate that Vitamin C metabolism and bioavailability may vary depending on sex, which can affect its efficacy in bone tissue protection. Consequently, differences in consumption may directly correlate with varying risk thresholds for men and women regarding the deterioration of bone structure [37]. Phytosterols, traditionally known for their ability to lower cholesterol levels, demonstrate a significant correlation with bone structure. Although the direct mechanisms of their action on bone are less explored, some recent research suggests that phytosterols may exert anti-inflammatory effects or influence signalling pathways associated with osteoblast differentiation [38,39]. The data obtained expand the spectrum of nutrients influencing bone structure, underscoring that skeletal health is the result of a complex interplay involving not only major macro- and micronutrients (calcium, protein, Vitamin D) but also other regulatory substances (selenium, iodine, Vitamin B6, phytosterols). The discovered associations are well aligned with contemporary understanding of bone metabolism and further complement it by highlighting the potential importance of components less traditionally associated with osteology.

Calcium and vitamin D deficiencies in older adults are associated with insufficient intake of foods rich in these nutrients, such as dairy products, fish (a source of calcium) and fatty fish, and eggs (a source of vitamin D). In addition, high consumption of sweets and caffeinated beverages may increase the risk of developing osteoporosis. This is due to a violation of calcium metabolism: excess sugar and caffeine contribute to an increase in the excretion of calcium in the urine and a decrease in its absorption in the intestine. A total of 71.7% of respondents showed a preference for strong tea. It should be noted that excessive consumption of strong tea can contribute to the leaching of useful nutrients from the body, which, in turn, exacerbates the lack of micronutrients. A study by Chinese scientists found that people who consumed less than 4.5 cups of tea a day had a lower risk of developing negative effects related to bone health [40]. However, taking into account national characteristics, Kazakhstanis on average consume about 7–8 mugs of tea per day, which significantly exceeds the specified threshold. Poor diets are not only associated with financial circumstances, but also a lack of education on rational nutrition and eating habits that affect the intake of macronutrients and macronutrients, such as tea traditions, a lack of greens, and the economy. Technological factors also impact the lack of nutrient intake in rare cases. Nutrition is also associated with a high risk of low-energy fractures among the senior gerontological population, where osteoporosis was detected in 19.4% of cases in the older working-age population.

BMI is a mandatory and integral risk factor utilized by the FRAX tool for the accurate assessment of the 10-year absolute risk of osteoporotic fractures. Our findings, which indicate a high average 10-year risk of major osteoporotic fractures in the studied Kazakhstani population (23.9 ± 0.65), underscore the seriousness of the epidemiological situation regarding osteoporosis in the region. This value significantly exceeds the intervention thresholds established in both national and international guidelines [41,42]. The high density distribution of fracture risk found among ethnic Kazakh individuals aged 60–89 years and the strong correlation of this risk with age (r = 730.673) are fully consistent with the global trend. Age is recognized as the most potent and independent predictor of osteoporotic fracture. In light of the aging population of Central Asia, these results are comparable to data obtained in other Asian countries where a sharp increase in the fracture burden is observed. Furthermore, a study conducted in Taldykorgan also highlights insufficient awareness and low screening coverage, despite the high population risk, which renders the FRAX tool, adapted to local conditions, critically important [43].

As a result of our study, we determined that the onset time (or age of manifestation) of osteoporosis varies depending on the BMI. Notably, respondents with low body weight develop osteoporosis on average at 65 years of age. At the same time, people with normal weight develop the disease later, on average at 69.47 years, those with overweight at 71.15 years, and those with obesity at 72.14 years.

The sign of age plays a significant role in the development of osteoporosis, as well as in the occurrence of excess body fat. In addition, there were significant differences (*p* < 0.001) between the indicators of body mass index depending on the state of bone tissue and the gender of respondents, where women in all groups had a higher BMI compared to men, and the frequency of detection of OP increased statistically significantly with weight loss (women—χ^2^ = 26.0, df = 2, *p* < 0.001; men—χ^2^ = 4.29, df = 2, *p* < 0.014; whole group—χ^2^ = 32.3, df = 2, *p* < 0.001).

Thus, the prevalence of osteoporosis significantly decreased with increasing BMI across both the overall group and the contingent of individuals classified as overweight and obese. This finding is consistent with extensive data from international systematic reviews and scientific studies that confirm the protective role of high BMI in relation to Bone Mineral Density (BMD) [44,45,46,47,48,49,50,51].

Violations of the strength properties of bone tissue with a decrease in body mass index and BMI were statistically significantly increased. Among the gerontological population of Kazakhstan, low body weight increases the risk of developing impaired bone strength by 2.24 times, and extreme morbid obesity also leads to a high risk of fractures. In another study, a lower BMI was found to increase the risk of developing osteoporosis by 8% [11]. These findings underscore the importance of maintaining an optimal BMI for bone health, particularly in individuals over 60 years of age.

The primary methodological limitations of the present cross-sectional study include the inherent difficulty in establishing direct cause-and-effect relationships between risk factors and the prevalence of osteoporosis, as well as the presence of gender imbalance in the sample (predominance of women). This imbalance is attributed not only to the demographic peculiarities of Kazakhstan’s elderly population but also to the factor of self-selection (voluntary participation), which may affect the external validity of the obtained data when extrapolated to the entire gerontological group of the region.

Our research is unique due to the primary assessment of the overall prevalence of osteoporosis among individuals aged over 60 years within a single geographical space—the Northern Region of Kazakhstan. The administrative units included (Akmola, Pavlodar regions, and the city of Astana) share similar climatic and demographic characteristics. This allowed for the study of the interrelationship between osteoporosis prevalence and alimentary factors within this specific population during the cross-sectional screening.

Considering that, osteoporosis and osteopenia remain a significant medical and social challenge, a promising direction for future work is the assessment of α-Klotho protein levels in the elderly residents of Kazakhstan. Studying the correlation of α-Klotho with BMD parameters and bone turnover markers will facilitate a deeper understanding of the molecular mechanisms underlying osteoporosis and improve strategies for healthy longevity.

## 8. Conclusions

This study makes a significant contribution to the literature as it presents the first screening data on calcaneal densitometry for the elderly population in the Northern Region of Kazakhstan, along with FRAX calculations conducted within the gerontological group of this population.

It is established that a higher BMI is associated with a lower incidence of osteoporosis, whereas low BMI and low body weight correlate with reduced BMD. These results underscore the critical importance of assessing both nutritional status and bone density when determining risks in the elderly population. The obtained data provide an evidence base for developing practical recommendations aimed at improving the Standard of Organization of Geriatric and Gerontological Care in the Republic of Kazakhstan dated June 23, 2021, №DSM-55. To optimize the prevention and early diagnosis of diseases in the elderly, it is recommended to introduce comprehensive screening programs that include regular monitoring of FRAX indicators: assessment of prenosological markers (nutritional status) and calcaneal densitometry, especially in respondents older than 60 years.

## Figures and Tables

**Table 1:** Actual consumption of essential nutrients that affects the development of osteoporosis among people over working age in Kazakhstan.

Nutritional Factors	Women	Men
Calorie content, (kcal)	1800.2 ± 242.3	1820.0 ± 322.2
Protein, (g)	70.2 ± 18.3	78.4 ± 32.5
Animal protein, (g)	37.8 ± 23.3	44.1 ± 29.3
Fiber (cellulose), (g)	5.7 ± 3.6	5.6 ± 3.4
Monounsaturated fatty acids (MUFAs), (g)	17.5 ± 9.5	19.8 ± 9.9
Phytosterol, (mg)	22.5 ± 37.0	23.9 ± 41.4
Sodium, (mg)	1191.0 ± 801.5	1541.3 ± 901.0
Potassium, (mg)	2681.1 ± 1252	2835.8 ± 1319.1
Calcium, (mg)	528.9 ± 308.2	549.6 ± 353.5
Magnesium, (mg)	249.6 ± 109.1	274.0 ± 111.4
Phosphorus, (mg)	918.2 ± 375	1019.0 ± 420.4
Zinc, (mg)	7.14 ± 4.06	8.51 ± 4.98
Selenium, (mcg)	35.5 ± 26.8	50.5 ± 44.5
Iodine, (mcg)	41.2 ± 32.6	44.1 ± 30.5
Manganese, (mg)	2.15 ± 1.18	2.55 ± 1.32
RE (retinol equivalent), mcg	682.8 ± 1536.2	739.2 ± 2068.9
Vitamin D, (mcg)	0.92 ± 1.71	1.15 ± 2.2
Vitamin C, (mg)	55.7 ± 53.9	52.5 ± 118.7
Pearson’s Correlation	R	Value
Calcium, (mg)	0.713 **	Correlation is significant at 0.01 (two-way)
Protein, total, (g)	0.538 **	
Phytosterol, (mg)	0.576 **	
Zinc, (mg)	0.575 **	
Selenium, (mcg)	0.630 **	
Iodine, (mcg)	0.609 **	
Vitamin B6, (mg)	0.630 **	
ANOVA	F	Meaning
Animal proteins, total, (g)	8.055	0.005
Nitrogen, (g)	7.254	0.008
Vitamin C, (mg)	6.904	0.010
Fiber (cellulose), (g)	6.358	0.013
Monounsaturated fatty acids (MUFAs), (g)	4.142	0.044
Vitamin B6, (mg)	3.735	0.056
RE (retinol equivalent), (mcg)	2.839	0.095

Note: ** *p* < 0.01.

**Table 2:** Proportion of people with deficient calcium and vitamin D intake by gender.

Studied	All Surveyed Respondents Were Studied	% of Individuals with a Consumption Level of Less than 2/3 RDA *
Insufficient calcium intake		
Whole group	1961 (100%)	1579 (80.5%)
Women	1619 (100%)	1305 (80.6%)
Men	342 (100%)	274 (80.1%)
Insufficient intake of vitamin D		
The whole group	1961 (100%)	1923 (98.1%)
Women	1619 (100%)	1593 (98.4%)
Men	342 (100%)	330 (96.5%)

Note: * RDAs—recommended dietary allowance.

**Table 3:** BMI depending on gender and age gradation and densitometry data (norm, OP, Osteopenia) for the surveyed respondents.

BMI, kg/m^2^		<18.5	18.5–24.99	25–29.99	30–34.99	35+	All	*p*
1	2	3	4	5	6	7	8	9
The entire group of respondents								
In general, *n*, %	of Norms	2 (8.3)	79 (16.1)	147 (19.6)	107 (23.9)	69 (27.7)	404 (20.6)	0.001
	Osteopenia	14 (58.3)	299 (60.9)	453 (60.4)	264 (59.1)	147 (59.0)	1177 (60.0)	
	OP	8 (33.3)	113 (23.0)	150 (20.0)	76 (17.0)	33 (13.3)	380 (19.4)	
	Total	24 (100.0)	491 (100.0)	750 (100.0)	447 (100.0)	249 (100.0)	1961 (100.0)	
Gender attribute								
Woman, *n*, %	of Norms	2 (11.1)	55 (14.4)	112 (18.4)	87 (22.9)	61 (26.3)	317 (19.6)	0.001
	Osteopenia	8 (44.4)	236 (61.8)	366 (60.2)	226 (59.5)	139 (59.9)	975 (60.2)	
	OP	8 (44.4)	91 (23.8)	130 (21.4)	67 (17.6)	32 (13.8)	328 (20.2)	
	Total	18 (100.0)	382 (100.0)	608 (100.0)	380 (100.0)	232 (100.0)	1620 (100.0)	
Men, *n*, %	of Norms	0	24 (22.0)	35 (24.6)	20 (29.9)	8 (47.1)	87 (25.5)	0.156
	Osteopenia	6 (100.0)	63 (57.8)	87 (61.3)	38 (56.7)	8 (47.1)	202 (59.2)	
	OP	0	22 (20.2)	20 (14.1)	9.0 (13.4)	1.0 (5.9)	52 (15.2)	
	Total	6 (100.0)	109 (100.0)	142 (100.0)	67 (100.0)	17 (100.0)	341 (100.0)	
Age, years								
60–69 years, *n*, %	of Norms	0	7.0 (10.0)	18 (11.6)	17 (16.5)	17 (25.4)	59 (14.8)	0.06
	Osteopenia	1.0 (33.3)	39 (55.7)	96 (61.9)	56 (54.4)	39 (58.2)	231 (58.0)	
	OP	2.0 (66.7)	24 (34.3)	41 (26.5)	30 (29.1)	11 (16.4)	108 (27.1)	
	Total	3 (100.0)	70 (100.0)	155 (100.0)	103 (100.0)	67 (100.0)	398 (100.0)	
70–79 years, *n*, %	of norms	0	3 (7.9)	8 (9.5)	4 (10.8)	7.0 (24.1)	22 (11.6)	0.033
	Osteopenia	0	14 (36.8)	34 (40.5)	23 (62.2)	14 (48.3)	85 (44.7)	
	OP	2 (100.0)	21 (55.3)	42 (50.0)	10 (27.0)	8.0 (27.6)	83 (43.7)	
	Total	2 (100.0)	38 (100.0)	84 (100.0)	37 (100.0)	29 (100.0)	190 (100)	
80–89 years, *n*, %	Osteopenia	0	1.0 (14.3)	1,0 (14.3)	4.0 (50.0)	0	6.0 (26.1)	0.291
	OP	0	6.0 (85.7)	6.0 (85.7)	4.0 (50.0)	1.0 (100.0)	17 (73.9)	
	Total	0	7.0 (100.0)	7.0 (100.0)	8.0 (100.0)	1.0 (100.0)	23 (100.0)	

**Table 4:** Body mass index of respondents depending on the state of bone tissue.

Respondents	BMI	
Norm	29.91 ± 3.92	R^R2^ = 0.9997
Osteopenia	28.57 ± 1.69	
Osteoporosis	27.76 ± 5.79	

**Table 5:** Body mass index of respondents depending on the state of bone tissue and gender.

Respondents	BMI		t	*p*
	Men	Women		
Norm	28.25 ± 3.51	30.36 ± 2.17	0.51	*p* > 0.05
Osteopenia	26.76 ± 2.02	28.94 ± 1.88	0.79	
Osteoporosis	26.02 ± 4.17	28.03 ± 2.08	0.43	

**Table 6:** Decrease in the detection of OP with an increase in BMI in respondents.

BMI	Osteoporosis				Violation of Bone Strength Properties (OP+ Osteopenia)			
	“+” *n* (%)	“−” *n* (%)	χ^2^ (df)	*p*	“+” *n* (%)	“−” *n* (%)	χ^2^ (df)	*p*
<18.5	8 (33.3)	16 (66.7)	14.9 (4)	<0.005	22 (91.7)	2 (8.3)	19.5 (4)	<0.001
18.5–24.9	113 (23)	378 (77)			412 (83.9)	79 (16.1)		
25–29.9	150 (20)	600 (80)			603 (80.4)	147 (19.6)		
30–34.9	76 (17)	371 (83)			340 (76.1)	107 (23.9)		
35+	33 (13.3)	216 (86.7)			180 (72.3)	69 (27.7)		

## Data Availability

The original contributions presented in this study are included in the article. Further inquiries can be directed to the corresponding author.

## Abbreviations

OP	Osteoporosis
BMI	Body Mass Index
BMD	Bone Mineral Density
25(OH)D	25-hydroxyvitamin D
DXA	Dual-energy X-ray Absorptiometry
WHO	World Health Organization
FAO	Food and Agriculture Organization of the United Nations
FRAX	Fracture Risk Assessment Tool
CI	Confidence Interval
SD	Standard Deviation
ANOVA	Analysis of Variance
IBM SPSS	Statistical Package for the Social Sciences
SOS	Speed of Sound
BUA	Broadband Ultrasound Attenuation
MUFA	Monounsaturated Fatty Acids
RE	Retinol Equivalent
LCB	Local Committee on Bioethics
